# Korean Red Ginseng Extract Inhibits IL-8 Expression via Nrf2 Activation in *Helicobacter pylori*-Infected Gastric Epithelial Cells

**DOI:** 10.3390/nu14051044

**Published:** 2022-02-28

**Authors:** Hae Sou Kim, Joo Weon Lim, Hyeyoung Kim

**Affiliations:** Department of Food and Nutrition, College of Human Ecology, Yonsei University, Seoul 03722, Korea; hime6409@naver.com (H.S.K.); jwlim1@yonsei.ac.kr (J.W.L.)

**Keywords:** gastric epithelial cells, *Helicobacter pylori*, interleukin-8, nuclear factor erythroid-2-related factor 2, *Panax ginseng*

## Abstract

*Helicobacter pylori* (*H. pylori*) causes gastric diseases by increasing reactive oxygen species (ROS) and interleukin (IL)-8 expression in gastric epithelial cells. ROS and inflammatory responses are regulated by the activation of nuclear factor erythroid-2-related factor 2 (Nrf2) and the expression of Nrf2 target genes, superoxide dismutase (*SOD*) and heme oxygenase-1 (*HO-1*). We previously demonstrated that Korean red ginseng extract (RGE) decreases *H. pylori*-induced increases in ROS and monocyte chemoattractant protein 1 in gastric epithelial cells. We determined whether RGE suppresses the expression of IL-8 via Nrf2 activation and the expression of *SOD* and *HO-1* in *H. pylori*-infected gastric epithelial AGS cells. *H. pylori*-infected cells were treated with RGE with or without ML385, an Nrf2 inhibitor, or zinc protoporphyrin (ZnPP), a HO-1 inhibitor. Levels of ROS and IL-8 expression; abundance of Keap1, HO-1, and SOD; levels of total, nuclear, and phosphorylated Nrf2; indices of mitochondrial dysfunction (reduction in mitochondrial membrane potential and ATP level); and SOD activity were determined. As a result, RGE disturbed Nrf2–Keap1 interactions and increased nuclear Nrf2 levels in uninfected cells. *H. pylori* infection decreased the protein levels of SOD-1 and HO-1, as well as SOD activity, which was reversed by RGE treatment. RGE reduced *H. pylori*-induced increases in ROS and IL-8 levels as well as mitochondrial dysfunction. ML385 or ZnPP reversed the inhibitory effect of RGE on the alterations caused by *H. pylori*. In conclusion, RGE suppressed IL-8 expression and mitochondrial dysfunction via Nrf2 activation, induction of SOD-1 and HO-1, and reduction of ROS in *H. pylori*-infected cells.

## 1. Introduction

*Helicobacter pylori* (*H. pylori*) infection is associated with atrophic gastritis, which can result in peptic ulcers or gastric cancer [1]. We previously showed that *H. pylori* induces the activation of nicotinamide adenine dinucleotide phosphate (NADPH) oxidase to produce reactive oxygen species (ROS) in gastric epithelial cells [2]. Oxidative stress contributes to the development of *H. pylori*-associated gastric diseases [3]. *H. pylori*-induced ROS cause an increase in the levels of inflammatory cytokines, including those of interleukin (IL)-8 [4]. IL-8 recruits inflammatory cells, neutrophils, and basophils to sites of infection, resulting in the propagation of inflammation. This can stimulate the subsequent production of ROS [5,6,7]. In addition, uncontrolled production of ROS can impair mitochondrial function [8].

Mitochondria are a major source of cellular energy. Furthermore, they are responsible for ROS production by cells. Overproduction of ROS causes mitochondrial dysfunction, resulting in increased mitochondrial ROS in damaged cells [9,10]. Mitochondrial dysfunction is strongly associated with the pathogenesis of oxidative stress-mediated injury [11]. Mitochondrial ROS act as signaling agents to stimulate inflammatory mediators and have been linked to the expression of pro-inflammatory cytokines [12,13].

Nuclear factor erythroid 2-related factor 2 (Nrf2) is a transcriptional regulator of antioxidant enzymes, including superoxide dismutase (SOD) and heme oxygenase-1 (HO-1) [14]. Nrf2 is a crucial factor in cellular defenses against oxidant-associated damage. Nrf2 is suppressed in the cytoplasm under normal conditions by anchoring to the redox sensor Kelch-like ECH-associated protein 1 (Keap1). Under inflammatory stimuli, interactions involving Nrf2 and Keap1 are disturbed, leading to the nuclear translocation of Nrf2. Phosphorylation of Nrf2 or modification of Keap1–SH groups activates the disruption of Nrf2–Keap1 complexes [14]. In the nucleus, Nrf2 binds to Maf proteins, stimulates antioxidant response elements (AREs), and induces the expression of antioxidant enzymes such as SOD-1 and HO-1 [15,16,17]. HO-1 produces antioxidants such as biliverdin/bilirubin and carbon monoxide by degrading heme [16,17]. SOD converts superoxide anions to oxygen and hydrogen peroxide [18,19]. Activation of Nrf2 exerts cellular defensive effects against oxidative injury and inflammation by upregulating cellular protective genes such as *HO-1* and *SOD-1* [20,21,22].

Korean ginseng (*Panax ginseng*) is a traditional herbal medicine used in East Asia. Red ginseng is harvested after 6 years, steamed, and then dried. Korean red ginseng has both anti-inflammatory and antioxidant effects. The major bioactive components of ginseng are ginsenosides composed of triterpene and sugar moieties [23]. In addition to ginsenosides, antioxidants such as polyacetylenes, acid polysaccharides, phenolic compounds, and alkaloids are present in ginseng and Korean red ginseng [24,25,26]. Previous studies have shown that Korean red ginseng extract (RGE) decreases inflammatory responses and apoptosis in *H. pylori*-infected gastric epithelial cells and gastric mucosa tissues of Mongolian gerbils [27,28,29]. Huang et al. [30] showed an inhibitory effect of ginsenosides on mitochondrial dysfunction. We previously showed that RGE prevents mitochondrial dysfunction by reducing intracellular and mitochondrial ROS in amyloid-β-treated human neuronal cells [31].

The aim of this study was to determine whether RGE inhibits the expression of IL-8 and mitochondrial dysfunction via Nrf2 activation and expression of SOD-1 and HO-1 in *H. pylori*-infected gastric epithelial AGS cells. To examine the association between Nrf2 and HO-1 and the protective effect of RGE against *H. pylori*-induced alterations, infected cells were treated with RGE in the presence or absence of ML385, an Nrf2 inhibitor, or zinc protoporphyrin (ZnPP), a HO-1 inhibitor.

## 2. Materials and Methods

### 2.1. Reagents, Cell Culture, and H. pylori Infection

RGE, a water extract of Korean red ginseng, was provided by the Korea Ginseng Corporation (Daejeon, Korea). RGE contains ginsenosides (7%) composed of Rb1 (8.27 mg/g), Rb2 (3.22 mg/g), Rc (3.90 mg/g), Rd (1.09 mg/g), Re (2.58 mg/g), Rf (1.61 mg/g), Rg1 (2.01 mg/g), (20S)-Rg2 (1.35 mg/g), (20S)-Rg3 (1.04 mg/g), and Rh1 (0.95 mg/g) [32]. ML385 was purchased from Sigma-Aldrich (St. Louis, MO, USA). ZnPP was purchased from Santa Cruz Biotechnology (Dallas, TX, USA). ZnPP and ML385 were dissolved in dimethyl sulfoxide (DMSO). For each experiment, the amount of vehicle was <0.1%. AGS cells were stimulated with *H. pylori* NCTC 11,637 at a ratio of 50:1 (bacteria:cells) [33].

### 2.2. Experimental Protocol

To determine the effect of RGE on the activation of Nrf2 (disturbance to Keap1–Nrf2 interaction and increased nuclear levels of Nrf2), AGS cells (7.5 × 10^5^/10 mL) were treated with RGE (1 μg/mL) and then stimulated with *H. pylori* for 1 h.

To determine the inhibitory effect of RGE on *H. pylori*-related alterations, cells (1.5 × 10^5^/2 mL/well or 7.5 × 10^5^/10 mL/dish) were pretreated with RGE (0.01, 0.1, and 1 μg/mL) for 1 h and stimulated with *H. pylori*. *H. pylori*-infected cells were cultured for 1 h (for assessment of mitochondrial and intracellular ROS levels; mitochondrial membrane potential (MMP); ATP levels; protein abundance of Keap1, HO-1, SOD-1, and SOD-2 and phospho-specific and total forms of Nrf2; and SOD activity), 4 h (for IL-8 mRNA determination), and 24 h (for assessing IL-8 levels in culture medium). To determine the involvement of Nrf2 and HO-1, the cells were treated with ML385 (5 μM) or ZnPP (2 μM) in the presence of RGE (1 μg/mL) for 1 h before *H. pylori* stimulation.

### 2.3. Real-Time PCR Analysis and Enzyme-Linked Immunosorbent Assay (ELISA) for IL-8

The expression of *IL-8* mRNA was determined using real-time PCR, following a previously described method [34]. An IL-8 ELISA kit (Catalog #D8000C, R&D Systems, Minneapolis, MN, USA) was used to measure IL-8 protein levels in the medium.

### 2.4. Measurement of Intracellular and Mitochondrial ROS Levels

Intracellular and mitochondrial ROS levels were determined by assessing the intensity of 2,7-dichlorofluorescein (DCF) and MitoSOX, following a previously described method [35].

### 2.5. Measurement of Mitochondrial Membrane Potential (MMP) and ATP Level

MMP abundance was measured by assessing the intensity of the fluorescence of JC-1 dye (green, excitation at 485 nm and emission at 535 nm; red, excitation at 590 nm and emission at 610 nm) [35]. The intensity ratio of red and green was assessed using ImageJ v.5.0 software (National Institutes of Health, Bethesda, MD, USA). An ATP assay kit (ab113849; Abcam, Cambridge, UK) was used to determine ATP levels [35].

### 2.6. Western Blotting

Whole-cell and nuclear extracts were prepared as previously described [35]. Western blot analysis was performed as described previously [29]. Briefly, cell extracts (10–40 μg/lane) were separated by 8–10% SDS polyacrylamide gel electrophoresis and electroblotting, and proteins were detected using antibodies against p-Nrf2 (ab76026, Abcam), Nrf2 (ab62352, Abcam), Keap1 (sc-365626, Santa Cruz Biotechnology), HO-1 (ADI-SPA-895-F, Enzo Life Sciences, NY, USA), SOD-1 (sc-11407, Santa Cruz Biotechnology), SOD-2 (sc-30080, Santa Cruz Biotechnology), lamin B1 (ab16048, Abcam), and β-actin (sc-47778, Santa Cruz Biotechnology). After detection with horseradish peroxidase-conjugated secondary antibody (anti-mouse or -rabbit), proteins were visualized using an enhanced chemiluminescence detection system (Santa Cruz Biotechnology) and an EZ-Capture ST imaging system (Atto, Tokyo, Japan). β-actin was used as the loading control. The densitometry data represent the mean ± standard error (SE) from three immunoblots and are shown as the relative density of protein bands normalized to the indicated protein (input Nrf2, input Keap1, actin, or lamin B1).

### 2.7. Immunoprecipitation of the Nrf2–Keap1 Complex

Nrf2–Keap1 interactions were determined by immunoprecipitation of Nrf2–Keap1 complexes, following a previously described method [36].

### 2.8. Assay for SOD Eznyme Activity

An SOD assay kit (BioVision, Inc., Milpitas, CA, USA) was used to measure SOD activity.

### 2.9. Statistical Analysis

All values are expressed as the mean ± standard error (SE) (*n* = 12 per group). Analysis of variance (ANOVA), followed by Tukey’s post hoc test, was used for the statistical analysis. A *p*-value of 0.05 or less was considered statistically significant.

## 3. Results

### 3.1. RGE Activates Nrf2 by Increasing Nuclear Translocation of Nrf2 and Decreasing Interactions between Nrf2 and Keap1 in AGS Cells

**Figure 1:**
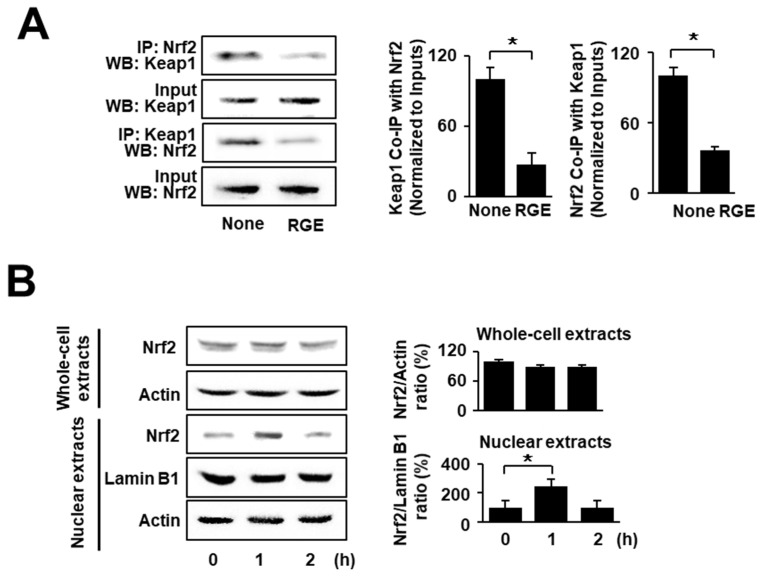
RGE suppresses the interaction of Nrf2 and Keap1 but increases nuclear level of Nrf2 in AGS cells. (**A**) The cells were treated with RGE (1 μg/mL) for 1 h. Western blots (WB) of whole-cell extracts (Input) and whole-cell extract-derived immunoprecipitates were obtained using the anti-Nrf2 and anti-Keap1 antibodies for immunoprecipitation (IP), and visualized, as indicated. “Input” is used as the control for protein expression (left panel). The ratios of Keap1 Co-IP with Nrf2 and Nrf2 Co-IP with Keap1 were determined from band densities of these proteins. None (cells without RGE treatment) was set at 100. * *p* < 0.05 (right panel). (**B**) The cells were treated with RGE (1 μg/mL) for the indicated time periods. The levels of Nrf2 in whole-cell extracts and nuclear extracts were examined by western blotting. Lamin B1 was used as a nuclear marker. Actin was used as a loading control (left panel). The ratios of Nrf2/Actin in whole-cell extracts and Nrf2/Lamin B1 in nuclear extracts were determined from band densities of these proteins. None (cells without RGE treatment) was set at 100. * *p* < 0.05 (right panel). The densitometry data represent the mean ± standard error (SE) from three immunoblots and are shown as the relative density of protein bands normalized to the indicated protein (input Nrf2, input Keap1, actin, or lamin B1). Co-IP, co-immunoprecipitation.

To determine the effects of RGE on Nrf2–Keap1 interactions and nuclear translocation of Nrf2 in uninfected cells, the cells were cultured for 1 h. The level of Nrf2–Keap1 association was determined by performing immunoprecipitation (IP) of the complexes with anti-Nrf2 or anti-Keap1 antibody followed by western blot analysis using anti-Keap1 and anti-Nrf2 antibody, respectively. As shown in Figure 1A, RGE slightly increased the levels of Keap1 and Nrf2 in whole-cell extracts (input; 2nd and 4th lines). Meanwhile, RGE increased the interaction involving Keap1 and Nrf2, since the levels of Nrf2 and Keap1 present after IP were decreased by treatment with RGE (IP; 1st and 3rd lines). Next, western blot analysis of whole-cell and nuclear extracts was performed using AGS cells treated with RGE for 1 and 2 h. As shown in Figure 1B, the level of Nrf2 in the nuclear extracts reached a maximum at 1 h and then decreased. These results show that RGE induced Nrf2 activation by suppressing Keap1-mediated sequestration of Nrf2 and facilitating its translocation into the nucleus.

### 3.2. RGE Suppresses Reduction in Phosphorylated Nrf2, HO-1, and SOD-1 Levels and SOD Activity in H. pylori-Infected AGS Cells

**Figure 2:**
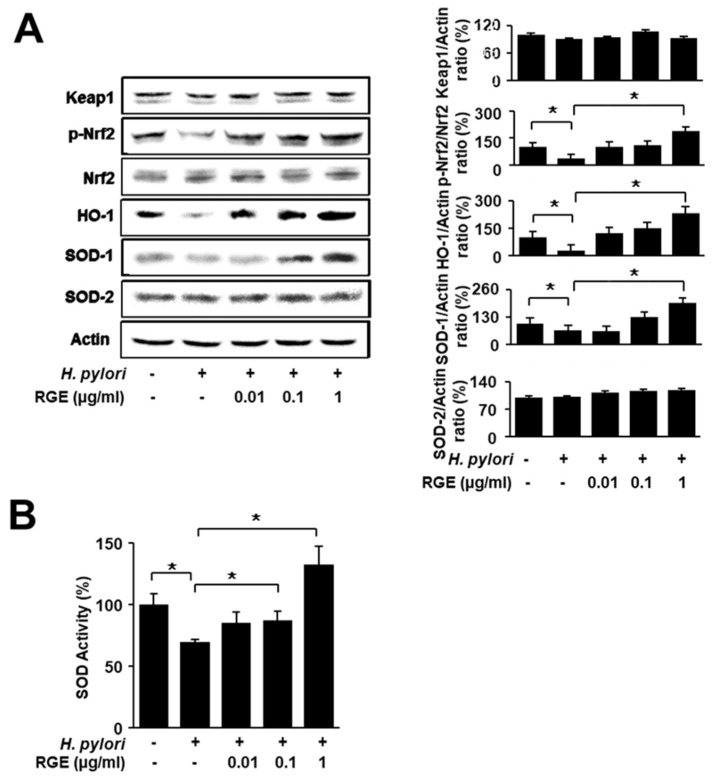
RGE suppresses reduction in phosphorylated Nrf2, HO-1, and SOD-1 levels and SOD activity in *H. pylori*-infected AGS cells. The cells were pretreated with the indicated concentrations of RGE for 1 h and then infected with *H. pylori* for 1 h. (**A**) The levels of Nrf2, phosphorylated Nrf2, Keap1, HO-1, SOD-1, and SOD-2 in whole-cell extracts were determined by western blotting (left panel). The ratios of Keap1/Actin, p-Nrf2/Nrf2, HO-1/Actin, SOD-1/Actin, and SOD-2/Actin were determined from band densities of these proteins. The protein band of the untreated and uninfected cells was set at 100. * *p* < 0.05 (right panel). The densitometry data represent the mean ± standard error (SE) from three immunoblots and are shown as the relative density of protein bands normalized to actin. (**B**) SOD activity in whole-cell extracts was measured using an SOD assay kit. The SOD activity in the untreated and uninfected cells was set at 100%. * *p* < 0.05.

Figure 2A shows that *H. pylori* infection decreased the phosphorylation of Nrf2 and protein levels of HO-1 and SOD-1, while total Nrf-2 and SOD-2 levels were not changed by *H. pylori*. RGE inhibited *H. pylori*-induced decreases in p-Nrf2, HO-1, and SOD-1 in a dose-dependent manner. The levels of p-Nrf2, HO-1, and SOD-1 in RGE-treated cells with *H. pylori* infection were higher than those in uninfected cells. SOD activity was decreased by *H. pylori*, which was restored by RGE in a dose-dependent manner (Figure 2B). These findings indicate that RGE induces the activation of Nrf2 and the expression of Nrf2 target enzymes HO-1 and SOD-1 in *H. pylori*-infected cells.

### 3.3. RGE Suppresses Increases in Levels of Intracellular and Mitochondrial ROS and IL-8 in H. pylori-Infected AGS Cells

**Figure 3:**
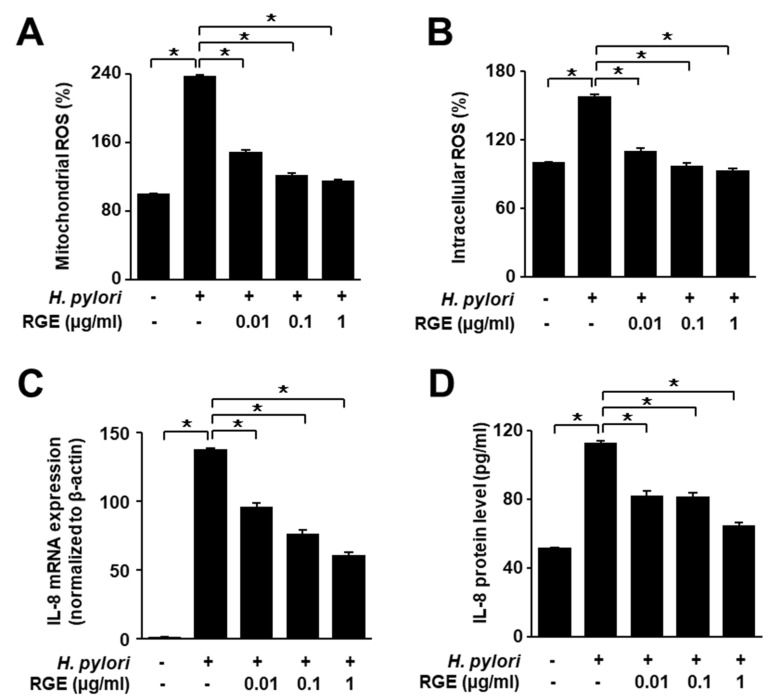
RGE suppresses the increases in ROS levels and IL-8 expression in *H. pylori*-infected AGS cells. The cells were pretreated with the indicated concentrations of RGE for 1 h and then infected with *H. pylori* for 1 h (**A**,**B**), 4 h (**C**), or 24 h (**D**). (**A**) Intracellular ROS was measured by dichlorofluorescein diacetate analysis. Intracellular ROS level in the untreated and uninfected cells was set at 100%. (**B**) Mitochondrial ROS was measured using MitoSOX. The mitochondrial ROS level in the untreated and uninfected cells was set at 100%. (**C**) IL-8 mRNA expression was quantified using real-time polymerase chain reaction (PCR) and normalized to β-actin. (**D**) IL-8 levels in the culture medium were assessed by enzyme-linked immunosorbent assay (ELISA). * *p* < 0.05.

*H. pylori* infection remarkably increased intracellular and mitochondrial ROS levels (Figure 3A,B). RGE suppressed *H. pylori*-induced increase in ROS levels in a dose-dependent manner. Real-time PCR and ELISA showed that *IL-8* mRNA and protein levels were both upregulated by *H. pylori* stimulation, which was suppressed by RGE (Figure 3C,D).

### 3.4. RGE Inhibits H. pylori-Induced Mitochondrial Dysfunction in AGS Cells

**Figure 4:**
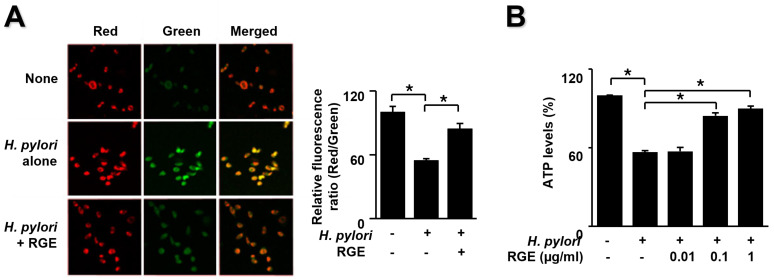
RGE suppresses mitochondrial dysfunction in *H. pylori*-stimulated AGS cells. The cells were pretreated with the indicated concentrations of RGE for 1 h and then infected with *H. pylori* for 1 h. (**A**) Mitochondrial membrane potential was assessed by staining JC-1 dye and red/green fluorescence images (left panel) and the ratio of red/green fluorescence (right panel). None, the untreated and uninfected cells; *H. pylori* alone, the untreated and *H. pylori*-infected cells; *H. pylori* + RGE, the RGE (1 μg/mL)-treated and *H. pylori*-infected cells. (**B**) ATP levels were assessed using an ATP assay kit. ATP level in the untreated and uninfected cells was set at 100%. * *p* < 0.05.

To determine the impact of RGE on *H. pylori*-mediated mitochondrial dysfunction, MMP and ATP levels in AGS cells were determined. We used the JC-1 fluorescence assay using a confocal microscope to observe images of untreated and uninfected cells (none), untreated and infected cells (*H. pylori* alone), and infected cells treated with 1 μg/mL RGE (*H. pylori* + RGE) (Figure 4A).

A membrane potential-sensitive color shift occurs by forming red fluorescent J-aggregates. Red/green fluorescence images are presented in Figure 4A (left panel). The red/green fluorescence ratios are shown in the right panel of Figure 3A. Membrane potentials are presented as the ratio of red/green fluorescence. *H. pylori* induced a decreased ratio of red/green fluorescence, which resulted in diminished MMP. RGE inhibited the decrease in red/green fluorescence ratio in *H. pylori*-stimulated cells. These results indicate that RGE suppresses the reduction in MMP in *H. pylori*-infected cells.

Figure 4B shows that *H. pylori* infection resulted in decreased ATP levels, which was reversed by RGE in a dose-dependent manner. These results demonstrate the preventive effect of RGE on mitochondrial dysfunction in *H. pylori*-infected cells. 

### 3.5. Nrf2 Inhibitor ML385 Inhibits the Effect of RGE on SOD-1 and HO-1 Levels in H. pylori-Infected AGS Cells

**Figure 5:**
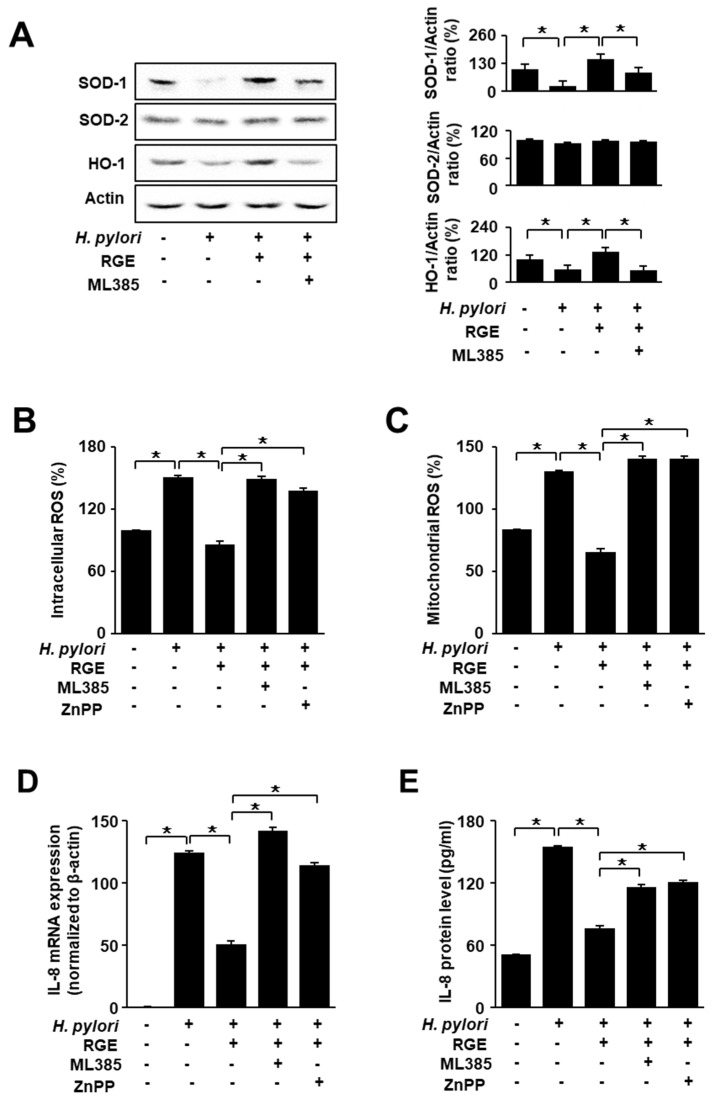
ML385 and ZnPP reverse the effect of RGE on the levels of SOD, HO-1, ROS, and IL-8 in *H. pylori*-stimulated AGS cells. The cells were co-treated with 1 μg/mL RGE and 2 μM ZnPP or 5 μM ML385 for 1 h, and then infected with *H. pylori* for 1 h (**A**–**C**), 4 h (**D**), or 24 h (**E**). (**A**) The levels of HO-1, SOD-1, and SOD-2 were determined by western blotting (left panel). The ratios of SOD-1/Actin, SOD-2/Actin, and HO-1/Actin were determined from band densities of these proteins. Control (cells without any treatment or stimulation) was set at 100. * *p* < 0.05 (right panel). (**B**) The level of intracellular ROS was measured by dichlorofluorescein diacetate analysis. The intracellular ROS level in the untreated and uninfected cells was set at 100%. (**C**) Mitochondrial ROS was measured using MitoSOX. The mitochondrial ROS level in the untreated and uninfected cells was set at 100%. (**D**) IL-8 mRNA expression was quantified using real-time polymerase chain reaction (PCR) and normalized to β-actin. (**E**) IL-8 levels in the culture medium were assessed by enzyme-linked immunosorbent assay (ELISA). * *p* < 0.05.

To confirm whether Nrf2 is associated with the inhibitory effect of RGE on *H. pylori*-induced reduction in SOD-1 and HO-1 levels, the Nrf2 inhibitor ML385 was added to RGE-treated infected cells. Figure 5A shows that RGE reversed the *H. pylori*-induced decreases in SOD-1 and HO-1, which were inhibited by ML385. SOD-2 levels were not affected by ML385 treatment. These results show that RGE increased the levels of HO-1 and SOD-1, which may be mediated by the activation of Nrf2 in *H. pylori*-stimulated cells.

### 3.6. Nrf2 Inhibitor ML385 and HO-1 Inhibitor ZnPP Attenuate the Inhibitory Effects of RGE on Increases in ROS and IL-8 Levels in H. pylori-Stimulated AGS Cells

To determine potential associations involving Nrf2 and HO-1, infected cells were treated with RGE containing an Nrf2 inhibitor, ML385, and a HO-1 inhibitor, ZnPP. Figure 5B,C shows that ML385 or ZnPP reversed the effect of RGE on reducing intracellular and mitochondrial ROS levels increased by *H. pylori*. As shown in Figure 5D,E, treatment with ML385 or ZnPP abolished the effect of RGE on IL-8 expression (at the mRNA and protein levels). These results suggest that Nrf2 and HO-1 function as mediators of the inhibitory effect of RGE on *H. pylori*-induced increases in ROS and IL-8 levels in AGS cells.

## 4. Discussion

Regarding pathological mechanisms, the involvement of oxidative stress, activation of mitogen-activated protein kinases, the Janus kinase-signal transducer and activator of transcription, and NF-κB in the induction of cytokines, including IL-8, has been demonstrated in *H. pylori*-infected gastric epithelial cells [34,37]. Since mitochondria are vulnerable to ROS and are also the major source of ROS [38], we determined whether *H. pylori* induces mitochondrial dysfunction, as determined by increased levels of mitochondrial ROS and reduced MMP and ATP production, in the present study. We demonstrated that increases in mitochondrial ROS were in parallel with reduced MMP and ATP levels in *H. pylori*-infected cells. Previously, we showed that RGE inhibits NADPH oxidase activity and reduces ROS production in *H. pylori*-infected cells [32]. Therefore, the antioxidant activity of RGE may prevent mitochondrial dysfunction and reduce mitochondrial ROS levels in *H. pylori*-infected cells, as shown in the present study. Since ROS activate inflammatory signaling to produce IL-8, RGE may inhibit IL-8 expression by reducing ROS (in whole cells and mitochondria) in *H. pylori*-stimulated cells.

Thirty-eight ginsenosides have been isolated from Korean ginseng (*Panax ginseng*) [39,40]. These ginsenosides have been divided into two groups based on structure—the panaxadiols (Rb1, Rb2, Rb3, Rc, Rd, Rg3, Rh2, and Rh3) and the panaxatriols (Re, Rf, Rg1, Rg2, and Rh1). RGE, used in the present study, contains ginsenosides such as Rb1, Rb2, Rc, Rd, Re, Rf, Rg1, (20S)-Rg2, (20S)-Rg3, and Rh1 [32] and other antioxidant components such as polyacetylenes, acidic polysaccharides, and phenolic compounds [24,25,26]. RGE and the isolated components of ginseng inhibited *H. pylori*-induced gastric diseases [41]. Rg3 induced the apoptosis of *H. pylori*-infected gastric cells by controlling the expression of Sp1 transcription factor and heat shock transcription factor 1 [42]. Protopanaxatriol and ginsenoside Rh1 did not suppress *H. pylori* growth [43]. However, panaxytriol inhibited *H. pylori* growth, while protopanaxatriol remarkably suppressed *H. pylori* urease and H^+^/K^+^ ATPase in the stomach, which are important factors in the treatment of gastric diseases [43,44]. In addition, acidic polysaccharides blocked *H. pylori* attachment to gastric epithelial cells in an in vitro experiment [45]. In a clinical setting, Korean red ginseng treatment showed beneficial effects on *H. pylori*-associated halitosis, chronic gastritis, and inflammation carcinogenesis [46,47,48]. These studies show the possible therapeutic use of RGE, a mixture of effective components of Korean red ginseng, for *H. pylori*-induced gastric diseases, including inflammation.

The present study investigated the role of Nrf2/HO-1, which has an inhibitory role against oxidative stress-induced tissue damage by stimulating antioxidant and phase 2 enzymes [49]. As Nrf2 target genes, SODs are a ubiquitous family of enzymes that catalyze the dismutation of superoxide anions. SOD-1, also called copper/zinc (Cu/Zn) SOD, is a key antioxidant enzyme that scavenges oxygen free radicals. SOD-2 has been localized to the mitochondria and uses manganese (Mn) as a cofactor. In contrast to SOD-1 and SOD-2, SOD-3 expression is restricted to only a few cell types in several tissues [50]. In the present study, we assessed the levels of SOD-1 and SOD-2 in *H. pylori*-infected cells treated with RGE. *H. pylori* infection decreased SOD-1 levels, which was reversed by RGE treatment. SOD-2 levels were not affected by *H. pylori* or RGE. These results suggest that RGE may induce SOD-1 to prevent oxidative stress-induced IL-8 expression via Nrf2 activation in AGS cells.

A previous study using Nrf2-deficient mice demonstrated that Nrf2 acts as a signaling mediator of SOD-1 expression in aged skeletal muscle [51]. Nrf2 knockdown prevented resveratrol-induced Nrf2 activation and reduced SOD-1 expression in porcine intestinal columnar epithelial IPEC-J2 cells [21]. In contrast, SOD-2 expression was found to be consistent. This study demonstrated that resveratrol increases SOD-1 expression through Nrf2 activation. These studies show that Nrf2 activation is an important protective mechanism against oxidative stress-induced cell injury by inducing SOD-1.

Kim et al. [52] showed that ginsenoside Rb2 induces transcriptional activation of the SOD-1 gene through the AP2 site. Chang et al. [53] demonstrated that the panaxadiol fraction and ginsenoside Rb2 could induce SOD-1, which is important for maintaining cell viability by reducing ROS in human hepatoma HepG2 cells. Rg1 and Rb1 showed neuroprotective effects against cerebral ischemia by inducing SOD-1 [54].

In this study, we used 0.01, 0.1, or 1 μg/mL of RGE. RGE (1 μg/mL) contains 8.27 ng/mL of Rb1, 3.22 ng/mL of Rb2, 3.90 ng/mL of Rc, and 1.09 ng/mL of Rd. Kim et al. [55] demonstrated that after single oral administration of RGE (9 g) in 10 healthy male Korean subjects, the plasma concentration of Rb1 was 3.94 ng/mL at 10 h and remained at this level at 40 h. Choi et al. [56] showed that after 15 days of repeated administration of RGE to 15 healthy Korean people, plasma concentrations of Rb1, Rb2, Rc, and Rd were 12.2, 8.83, 2.08, and 3.14 ng/mL, respectively. Based on these studies, the concentrations of RGE used in the present study are clinically relevant. 

Regarding the cell viability after treatment with *H. pylori* or RGE, previously we showed that the viable cell number of *H. pylori*-infected cells was 3 times higher than that of the uninfected cells at 24 h culture with an infection ratio of 50:1 (bacterium:cell) [33]. However, *H. pylori* at high concentration (at a ratio of 300:1, bacterium:cell) induced apoptosis, which was inhibited by RGE (0.01, 0.1, and 1 μg/mL) in a dose-dependent manner at 24 h culture [29]. A low infection ratio of *H. pylori* increased ROS levels, which may activate inflammatory signaling pathways to induce inflammatory cytokines such as IL-8 and interferon-γ in gastric mucosal tissues of animals and gastric epithelial AGS cells [28,57,58]. Six-week dietary supplementation of RGE did not affect *H. pylori* colonization and cell numbers of *H. pylori* in the stomachs of Mongolian gerbils [28]. The study suggests that RGE did not affect the colonization and viability of *H. pylori*. However, excess amounts of ROS, which are produced by a high infection ratio of *H. pylori*, induce cell death and oxidative DNA damage [29]. Since RGE reduces ROS levels and prevents ROS-mediated DNA damage, RGE inhibited *H. pylori*-induced apoptosis [29]. Therefore, *H. pylori* infection at the ratio of 50:1 (bacterium:cell), used in the present study, did not induce cell death but did increase proliferation, which is critical for carcinogenesis.

Regarding the importance of the present study in relation to animal experiments and future study, previously we found that *H. pylori* inoculation induced hyperplasia and inflammation of gastric mucosa of Mongolian gerbils [28]. The levels of IL-8 and lipid peroxides and myeloperoxidase activity were higher in gastric mucosa of the infected animals than those of uninfected animals. Dietary supplementation of RGE (200 mg RGE/gerbil) prevented gastric hyperplasia and inflammation by decreasing the levels of IL-8 and oxidative stress indices of gastric mucosal tissues [28]. The aim of the present study was to determine the underlying mechanism of how RGE reduces ROS and IL-8 levels by using an in vitro infected cells model. We here found that RGE disrupts the interaction between Nrf2 and Keap1, resulting in nuclear translocation of Nrf2 in AGS cells. Therefore, RGE induces the expression of Nrf2 target genes SOD-1 and HO-1, which reduces ROS levels and IL-8 expression in *H. pylori*-infected cells. Activation of Nrf2 and induction of HO-1 and SOD-1 may contribute to the antioxidant mechanism of RGE. For further study, it should be determined whether dietary supplementation of RGE induces activation of Nrf2 and expression of HO-1 and SOD-1 in gastric mucosal tissues of *H. pylori*-infected animals.

**Figure 6:**
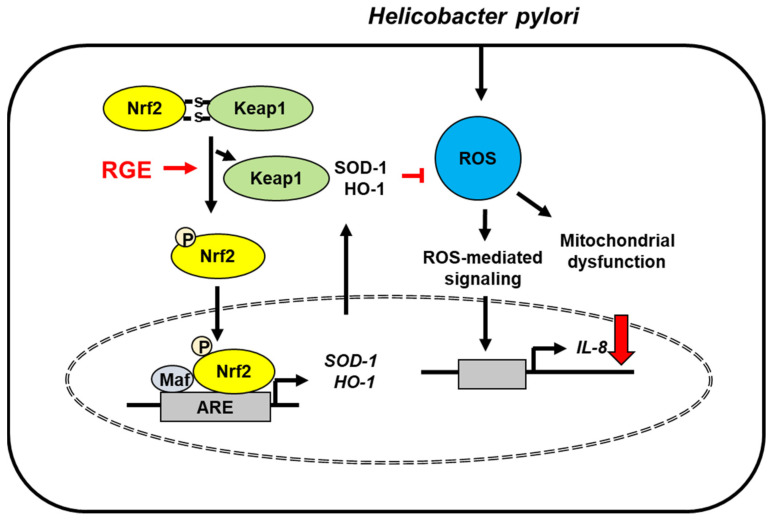
The proposed mechanism underlying the inhibitory effect of RGE on IL-8 expression in *Helicobacter pylori*-stimulated gastric epithelial cells. *Helicobacter pylori* increases ROS levels, which induces mitochondrial dysfunction and reactive oxygen species (ROS)-mediated signaling, leading to IL-8 expression. RGE disrupts the interaction between Nrf2 and Keap1, resulting in phosphorylation and nuclear translocation of Nrf2 in AGS cells. Therefore, RGE induces the expression of Nrf2 target genes SOD-1 and HO-1, which reduces ROS levels and IL-8 expression in *Helicobacter pylori*-infected cells. HO-1, heme oxygenase-1; IL-8, interleukin-8; Keap 1, kelch-like ECH-associating protein 1; Nrf-2, nuclear factor erythroid-2-related factor 2; RGE, red ginseng extract; ROS, reactive oxygen species; SOD-1, superoxide dismutase-1; stimulate (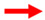); decrease (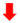); inhibit (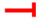).

The present study supports the beneficial effect of RGE against *H. pylori*-mediated gastric inflammation. As summarized in Figure 6, RGE induces the activation of Nrf2/HO-1 by disturbing the interaction between Nrf2 and Keap1 and facilitating nuclear translocation of Nrf2 in gastric epithelial cells. Nrf2 activation induces by RGE stimulates the expression of its downstream target antioxidant enzymes, such as SOD-1 and HO-1, thereby reducing ROS levels. *H. pylori* increases levels of ROS, which induces mitochondrial dysfunction and activates ROS-mediated signaling to induce IL-8 expression. RGE reduces ROS levels and inhibits mitochondrial dysfunction via Nrf2 activation and induction of HO-1 and SOD-1 in gastric epithelial cells. 

## 5. Conclusions

RGE suppresses IL-8 expression and mitochondrial dysfunction via Nrf2 activation, induction of SOD-1 and HO-1, and reduction of ROS in *H. pylori*-infected cells. Therefore, supplementation with RGE may exert beneficial effects in preventing *H. pylori*-induced gastric inflammation.

## Data Availability

The data used to support the findings of this study are included within the article.
